# Supplementary material for: Interventions for combating COVID-19 misinformation: A systematic realist review
Source: PLoS One. 2025 Apr 24;20(4):e0321818. doi: 10.1371/journal.pone.0321818 (PMC12021165; doi:10.1371/journal.pone.0321818)
Supplement: S2 File — (PDF) [file pone.0321818.s002.pdf]

Systematic review

A list of fields that can be edited in an update can be found here

1. * Review title.

Give the title of the review in English

Realist review: assessing intervention effectiveness in combating COVID misinformation

2. Original language title.

For reviews in languages other than English, give the title in the original language. This will be displayed with the English language title.

3. * Anticipated or actual start date.

Give the date the systematic review started or is expected to start.

01/07/2023

4. * Anticipated completion date.

Give the date by which the review is expected to be completed.

31/08/2024

5. * Stage of review at time of this submission.

This field uses answers to initial screening questions. It cannot be edited until after registration.

Tick the boxes to show which review tasks have been started and which have been completed.

Update this field each time any amendments are made to a published record.

The review has not yet started: Yes

Review stage	Started	Completed
Preliminary searches	No	No
Piloting of the study selection process	No	No
Formal screening of search results against eligibility criteria	No	No
Data extraction	No	No
Risk of bias (quality) assessment	No	No
Data analysis	No	No

Provide any other relevant information about the stage of the review here.

6. * Named contact.

The named contact is the guarantor for the accuracy of the information in the register record. This may be any member of the review team.

Robert Dickinson

Email salutation (e.g. "Dr Smith" or "Joanne") for correspondence:

Mr Dickinson

7. * Named contact email.

Give the electronic email address of the named contact.

robaldickinson@gmail.com

8. Named contact address

Give the full institutional/organisational postal address for the named contact.

Room 104 Watson Building, Brighton BN1 9BL

9. Named contact phone number.

Give the telephone number for the named contact, including international dialling code.

+44 (0)7380667211

10. * Organisational affiliation of the review.

Full title of the organisational affiliations for this review and website address if available. This field may be

completed as 'None' if the review is not affiliated to any organisation.

Brighton and Sussex Medical School

Organisation web address:

https://www.bsms.ac.uk

11. * Review team members and their organisational affiliations.

Give the personal details and the organisational affiliations of each member of the review team. Affiliation refers to groups or organisations to which review team members belong. **NOTE: email and country now MUST be entered for each person, unless you are amending a published record. PLEASE USE AN INSTITUTIONAL EMAIL ADDRESS IF POSSIBLE.**

Mr Robert Dickinson. University of Sussex
Dr Elizabeth Ford. Brighton and Sussex Medical School
Professor Harm van Marwijk. Brighton and Sussex Medical School

12. * Funding sources/sponsors.

Details of the individuals, organizations, groups, companies or other legal entities who have funded or sponsored the review.

Self-funded

Grant number(s)

State the funder, grant or award number and the date of award

13. * Conflicts of interest.

List actual or perceived conflicts of interest (financial or academic).

None

14. Collaborators.

Give the name and affiliation of any individuals or organisations who are working on the review but who are not listed as review team members. **NOTE: email and country must be completed for each person, unless you are amending a published record.**

Dr Dominique Makowski. University of Sussex

15. * Review question.

State the review question(s) clearly and precisely. It may be appropriate to break very broad questions down into a series of related more specific questions. Questions may be framed or refined using PI(E)COS or similar where relevant.

Which interventions are most effective in combating spread of and belief in COVID misinformation?

Sub-questions:

Which types of interventions work best?

Which groups of people do they work best for? (including sub-analysis of vulnerable communities)

Under what circumstances do the interventions work?

Which theories best explain how these interventions work?

What is the quality of studies testing interventions to combat spread of and belief in COVID misinformation?

16. * Searches.

State the sources that will be searched (e.g. Medline). Give the search dates, and any restrictions (e.g. language or publication date). Do NOT enter the full search strategy (it may be provided as a link or attachment below.)

Scopus

Web of Science

ASSIA

PsycINFO

PubMed

Publication dates: 2020-2023

Language: English

17. URL to search strategy.

Upload a file with your search strategy, or an example of a search strategy for a specific database, (including the keywords) in pdf or word format. In doing so you are consenting to the file being made publicly accessible. Or provide a URL or link to the strategy. Do NOT provide links to your search **results**.

Alternatively, upload your search strategy to CRD in pdf format. Please note that by doing so you are consenting to the file being made publicly accessible.

Yes I give permission for this file to be made publicly available

18. * Condition or domain being studied.

Give a short description of the disease, condition or healthcare domain being studied in your systematic review.

COVID-19 belief in or propagation of misinformation about Covid.

19. * Participants/population.

Specify the participants or populations being studied in the review. The preferred format includes details of both inclusion and exclusion criteria.

Inclusion: Adults and the elderly

Exclusion: Children and adolescents (under 18 years of age)

20. * Intervention(s), exposure(s).

Give full and clear descriptions or definitions of the interventions or the exposures to be reviewed. The preferred format includes details of both inclusion and exclusion criteria.

This review will include interventions that aim to reduce spread or or belief in misinformation related to COVID-19. These interventions include but are not limited to:

Fact Checking: either automated attempts to fact check in real-time or coming after the initial misleading information is published, either by the original institution publishing the misinformation or by independent fact-checkers. The intention here is that those who initially read the misinformation will also see this fact-check and change their beliefs accordingly.

Accuracy nudges: prompts that appear when an individual goes to read something marked as either misleading or a topic with high levels of misinformation, suggesting to the reader that they consider accuracy of the information and trust in the source.

Games: gamified interventions to teach about how misinformation is created and spread. In these games, the participants take the role of a misinformer, purposefully attempting to create misinformation and spread it as widely as possible. These take place separately from individual media consumption habits.

Education: generalised attempts to educate the public about digital literacy, misinformation, media sources, and bias, with the goal of improving digital literacy and giving participants a greater understanding of and resilience to misinformation in the future.

21. * Comparator(s)/control.

Where relevant, give details of the alternatives against which the intervention/exposure will be compared (e.g. another intervention or a non-exposed control group). The preferred format includes details of both inclusion and exclusion criteria.

Studies with and without control groups who did not receive the intervention will be included. However, those studies with a control group will be highlighted as part of outlining the robustness of each intervention's evidence.

22. * Types of study to be included.

Give details of the study designs (e.g. RCT) that are eligible for inclusion in the review. The preferred format includes both inclusion and exclusion criteria. If there are no restrictions on the types of study, this should be stated.

Randomised and quasi-randomised trials

Single-arm pre-post studies which measure outcomes before and after the intervention

Studies which provide an intervention and evaluate its effects via qualitative methods

23. Context.

Give summary details of the setting or other relevant characteristics, which help define the inclusion or exclusion criteria.

All interventions delivered online via the internet, with participants recruited through a variety of online mechanisms (e.g. MTurk).

24. * Main outcome(s).

Give the pre-specified main (most important) outcomes of the review, including details of how the outcome is defined and measured and when these measurement are made, if these are part of the review inclusion criteria.

To determine the efficacy of the interventions, outcomes to be measured include but are not limited to:

Participant self-reported willingness to share misinformation, either measured post-intervention or preferably pre- and post-intervention

Vulnerability to believing misinformation as measured within study-designs

Participant capacity to correct previously believed misinformation as measured within study-designs

Participant digital literacy in identifying trustworthy sources, avoiding misinformation, and identifying misinformation in their day-to-day lives, either measured after the intervention or preferably pre- and post-intervention

Qualitative first person accounts of belief or behaviour change following the intervention.

Measures of effect

Please specify the effect measure(s) for you main outcome(s) e.g. relative risks, odds ratios, risk difference, and/or 'number needed to treat.

25. * Additional outcome(s).

List the pre-specified additional outcomes of the review, with a similar level of detail to that required for main outcomes. Where there are no additional outcomes please state ‘None’ or ‘Not applicable’ as appropriate to the review

None

Measures of effect

Please specify the effect measure(s) for you additional outcome(s) e.g. relative risks, odds ratios, risk difference, and/or 'number needed to treat.

26. * Data extraction (selection and coding).

Describe how studies will be selected for inclusion. State what data will be extracted or obtained. State how this will be done and recorded.

Study selection: One reviewer will screen for inclusion, with team members checking their decisions. A second reviewer will duplicate some of the first reviewer's work for quality control purposes (10%). Duplicated work will be checked="checked" value="1" for inter-rater agreement. If this is low, the need to refine eligibility criteria will be assessed and a further 10% double-screened. Disagreements between judgments will be resolved through team discussion, with final say going to the reviewer.

Data extraction: Data to be extracted includes theoretical contributions and suggested frameworks for studying misinformation, conclusions regarding the appeal and spread of misinformation, and discussion of how each study fits within the broader literature on the topic. One reviewer will extract the data, with team members checking the data. Disagreements between judgments will be resolved through team discussion, with final say going to the reviewer. Missing data will be excluded from analysis. Study investigators of studies missing data will not be contacted. Data will be recorded through OneNote, Excel, and NVivo.

Coding: Coding will be done iteratively to categorise the findings. This iterative process will evolve into a developed scheme as the coding takes place. For instance, if one particular intervention was identified from an article during coding, the coder would try to assign it to a category within the intervention framework. New subcategories would be created if the current categories are insufficient, until all interventions are categorised. The same process will happen with theoretical explanations present within the reviewed articles. A second coder will follow the same procedure in a second round of coding to validate the scheme. Discrepancies to be resolved with discussion.

27. * Risk of bias (quality) assessment.

State which characteristics of the studies will be assessed and/or any formal risk of bias/quality assessment tools that will be used.

This review will utilise Kenny et al.'s (2019) risk of bias tool for assessing study rigor. https://systematicreviewsjournal.biomedcentral.com/articles/10.1186/s13643-018-0925-0 It includes eight items for appraisal: (1) cohort, (2) control or comparison group, (3) pre-post intervention data, (4) random assignment of participants to the intervention, (5) random selection of participants for assessment, (6) follow-up rate of 80% or more, (7) comparison groups equivalent on sociodemographics, and (8) comparison groups equivalent at baseline on outcome measures.

28. * Strategy for data synthesis.

Describe the methods you plan to use to synthesise data. This **must not be generic text** but should be **specific to your review** and describe how the proposed approach will be applied to your data. If meta-analysis is planned, describe the models to be used, methods to explore statistical heterogeneity, and software package to be used.

The strategy for data synthesis for this project centres on a realist review approach. This approach is relatively new, focusing on understanding the mechanisms by which an intervention works or fails on a certain outcome, in a certain context. Similar to a traditional systematic review, realist reviews appraise evidence with the explicit purpose of refining theory and using that theory to inform policy (Rycroft-Malone et al. 2012). This appraisal is iterative and continually informed by the derivation and refinement of theory taking place during the analysis of the included studies. Finally, the findings then explain why or why not (and in which contexts) the interventions work, enabling informed choices about policy, intervention, and further research. In this way, realist learning is intended to be highly transferable via the intervention mechanisms studied and the way context and theory are built into the analysis (Wong 2018).

The analysis will be performed as follows: First, all papers will be loaded into NVivo (A qualitative data analysis software). Second, the results, discussion, and conclusion sections will be read line by line. During this process, iterative coding and code-creation will simultaneously occur as described under Data Extraction (number 26). Third, codes will undergo duplication and validation as described above by a second coder. Fourth, codes will be organised into related areas, thereby creating descriptive themes, likely in a tree structure connecting codes hierarchically into broader themes. Fifth, the review will go beyond the findings of the included studies to produce a synthesis directly addressing the research questions of this review. Researcher inference alongside discussion with the second coder will allow for the emergence of analytical themes identifying barriers, facilitators, and implications for the interventions accomplishing their outcomes, which will be examined taking into account the descriptive themes. This process is cyclical, and will continue until the analytical themes sufficiently explain all descriptive themes as well as the barriers, facilitators, and implications previously mentioned. Sixth, the analytical themes will then inform the development of recommendations for interventions. For those papers with quantitative results, results will be grouped by intervention and outcome, then tabulated to identify patterns across the included quantitative studies, then thematically analysed alongside the qualitative studies to identify areas of commonality for synthesis.

29. * Analysis of subgroups or subsets.

State any planned investigation of ‘subgroups’. Be clear and specific about which type of study or participant will be included in each group or covariate investigated. State the planned analytic approach.

None planned.

30. * Type and method of review.

Select the type of review, review method and health area from the lists below.

Type of review

Cost effectiveness

No

Diagnostic
No

Epidemiologic
No

Individual patient data (IPD) meta-analysis
No

Intervention
Yes

Living systematic review
No

Meta-analysis
No

Methodology
No

Narrative synthesis
No

Network meta-analysis
No

Pre-clinical
No

Prevention
No

Prognostic
No

Prospective meta-analysis (PMA)
No

Review of reviews
No

Service delivery
No

Synthesis of qualitative studies
No

Systematic review
Yes

Other
No

Realist review

Health area of the review

Alcohol/substance misuse/abuse

No

Blood and immune system

No

Cancer

No

Cardiovascular

No

Care of the elderly

No

Child health

No

Complementary therapies

No

COVID-19

Yes

For COVID-19 registrations please tick all categories that apply. Doing so will enable your record to appear in area-specific searches

Chinese medicine
Diagnosis
Epidemiological
Genetics
Health impacts
Immunity
Long COVID
Mental health
PPE
Prognosis
Public health intervention
Rehabilitation
Service delivery
Transmission
Treatments
Vaccines
Other

Crime and justice

No

Dental

No

Digestive system

No

Ear, nose and throat

No

Education

No

Endocrine and metabolic disorders

No

Eye disorders

No

General interest

No

Genetics

No

Health inequalities/health equity

No

Infections and infestations

Yes

International development

No

Mental health and behavioural conditions

No

Musculoskeletal

No

Neurological

No

Nursing

No

Obstetrics and gynaecology

No

Oral health

No

Palliative care

No

Perioperative care

No

Physiotherapy

No

Pregnancy and childbirth

No

Public health (including social determinants of health)

Yes

Rehabilitation

No

Respiratory disorders

No

Service delivery

No

Skin disorders

No

Social care

Yes

Surgery

No

Tropical Medicine

No

Urological

No

Wounds, injuries and accidents

No

Violence and abuse

No

31. Language.

Select each language individually to add it to the list below, use the bin icon to remove any added in error.

English

There is not an English language summary

32. * Country.

Select the country in which the review is being carried out. For multi-national collaborations select all the countries involved.

England

33. Other registration details.

Name any other organisation where the systematic review title or protocol is registered (e.g. Campbell, or The Joanna Briggs Institute) together with any unique identification number assigned by them. If extracted data will be stored and made available through a repository such as the Systematic Review Data Repository (SRDR), details and a link should be included here. If none, leave blank.

34. Reference and/or URL for published protocol.

If the protocol for this review is published provide details (authors, title and journal details, preferably in Vancouver format)

Add web link to the published protocol.

https://docs.google.com/document/d/1-JFeHUYxzkzhaUQYRtC_IyKVKnJ9qrR3HZI44RdZezo/edit?usp=sharing

Or, upload your published protocol here in pdf format. Note that the upload will be publicly accessible.

No I do not make this file publicly available until the review is complete

Please note that the information required in the PROSPERO registration form must be completed in full even if access to a protocol is given.

35. Dissemination plans.

Do you intend to publish the review on completion?

Yes

Give brief details of plans for communicating review findings.?

in addition to producing a chapter for a PhD thesis at Brighton and Sussex Medical School which will be made available free of charge on the University website and a variety of PhD thesis archives, a paper will be submitted to a leading journal in this field to further disseminate the results of this review (e.g. JMIR). Furthermore, should the derived synthetical theory of misinformation warrant a change in practice, a one page summary report will be prepared and sent to healthcare professionals and policymakers involved in the US public health apparatus.

36. Keywords.

Give words or phrases that best describe the review. Separate keywords with a semicolon or new line.

Keywords help PROSPERO users find your review (keywords do not appear in the public record but are included in searches). Be as specific and precise as possible. Avoid acronyms and abbreviations unless these are in wide use.

Systematic review

Realist review

Meta-analysis

Thematic analysis

Misinformation

COVID-19

Conspiracy theories

Intervention

37. Details of any existing review of the same topic by the same authors.

If you are registering an update of an existing review give details of the earlier versions and include a full bibliographic reference, if available.

38. * Current review status.

Update review status when the review is completed and when it is published.New registrations must be ongoing so this field is not editable for initial submission.

Please provide anticipated publication date

Review_Ongoing

39. Any additional information.

Provide any other information relevant to the registration of this review.

This review is occurring as part of a PhD candidacy at Brighton and Sussex Medical School to inform the other analytical projects within the PhD candidacy.

40. Details of final report/publication(s) or preprints if available.

Leave empty until publication details are available OR you have a link to a preprint (NOTE: this field is not editable for initial submission). List authors, title and journal details preferably in Vancouver format.

Give the link to the published review or preprint.
