# Supplementary material for: Interventions for combating COVID-19 misinformation: A systematic realist review
Source: PLoS One. 2025 Apr 24;20(4):e0321818. doi: 10.1371/journal.pone.0321818 (PMC12021165; doi:10.1371/journal.pone.0321818)
Supplement: S1 Table — (DOCX) [file pone.0321818.s003.docx]

| **Article** | **Cohort** | **Control or comparison group** | **Pre-post intervention data** | **Random assignment of participants to the intervention** | **Random selection of participants for assessment** | **Follow-up rate 80% or higher** | **Comparison groups equivalent on demographics** | **Comparison groups equivalent at baseline on outcome measures** | **Total score (out of 8)** |
| --- | --- | --- | --- | --- | --- | --- | --- | --- | --- |
| Abroms et al. 2024 | Yes | Yes | Yes | Yes | Yes | No | Yes | Yes | 7 |
| Agley et al. 2021 | Yes | Yes | Yes | Yes | Yes | N/A | Yes | Yes | 7 |
| Amazeen et al. 2022 | Yes | Yes | Yes | Yes | Yes | N/A | Yes | N/A | 6 |
| Amin et al. 2021 | Yes | No | Yes | N/A | no | Yes | N/A | N/A | 3 |
| Armand et al. 2024 | Yes | Yes | Yes | Yes | Yes | No | Yes | Yes | 7 |
| Arechar et al. 2023 | Yes | Yes | Yes | Yes | Yes | No | Yes | Yes | 7 |
| Aslett et al. 2022 | Yes | Yes | Yes | Yes | Yes | Yes | Yes | N/A | 7 |
| Basol et al. 2020 | Yes | Yes | Yes | Yes | Yes | N/A | Yes | no | 6 |
| Bender et al. 2023 | Yes | Yes | No | Yes | no | N/A | Yes | no | 4 |
| Cook et al. 2024 | Yes | Yes | Yes | Yes | Yes | Yes | Yes | Yes | 8 |
| DeGarmo et al. 2022 | Yes | Yes | No | Yes | Yes | N/A | Yes | Yes | 6 |
| Dias et al. 2020 | Yes | Yes | Yes | Yes | Yes | N/A | Unknown | No | 5 |
| Freeman et al. 2021 | Yes | Yes | No | Yes | Yes | N/A | Yes | Yes | 6 |
| Fung et al. 2022 | Yes | No | Yes | Yes | no | Yes | N/A | N/A | 4 |
| Gavin et al. 2022 | No | Yes | No | Yes | Yes | N/A | Yes | N/A | 4 |
| Iles et al. 2022 | Yes | Yes | Yes | Yes | Yes | Yes | Yes | Yes | 8 |
| Jiang et al. 2022 | Yes | Yes | No | Yes | no | Yes | Yes | Yes | 7 |
| Johnson et al. 2022 | Yes | Yes | No | Yes | Yes | Yes | Yes | Unknown | 6 |
| Kreps et al. 2022 | Yes | Yes | No | Yes | Yes | N/A | Yes | N/A | 5 |
| Li et al. 2024 | Yes | Yes | Yes | Yes | Yes | Yes | Yes | Yes | 8 |
| Ma et al. 2023 | Yes | Yes | Yes | Yes | no | no | Unknown | Unknown | 4 |
| Maertens et al. 2021 | Yes | Yes | Yes | Yes | Yes | Yes | Yes | Yes | 8 |
| Maragh-Bass et al. 2022 | Yes | No | Yes | N/A | no | Yes | N/A | N/A | 3 |
| Msunyaro et al. 2023 | Yes | No | Yes | No | No | N/A | N/A | N/A | 2 |
| Offer-Westort et al. 2023 | Yes | Yes | Yes | Yes | Yes | Yes | Yes | Yes | 8 |
| Pennycook et al. 2020 | Yes | Yes | No | Yes | Yes | Yes | Yes | Unknown | 7 |
| Piltch-Loeb et al. 2022 | Yes | Yes | Yes | No | Yes | N/A | Yes | No | 5 |
| Rasmussen et al. 2022 | Yes | Yes | Yes | Yes | Yes | Yes | Yes | Yes | 8 |
| Spalvins et al. 2024 | Yes | Yes | Yes | Yes | Yes | Yes | Yes | Yes | 8 |
| Stekelenburg et al. 2021 | Yes | Yes | Yes | Yes | Yes | Yes | Yes | N/A | 7 |
| Ugarte et al. 2023 | Yes | Yes | No | Yes | Yes | Yes | Yes | N/A | 6 |
| Vandormael et al 2021 | Yes | Yes | Yes | Yes | Yes | N/A | Yes | Yes | 7 |
| Veletsianos et al. 2022 | Yes | No | No | N/A | N/A | N/A | N/A | N/A | 1 |
| Vijaykumar et al. 2021 | Yes | Yes | No | Yes | Yes | N/A | Yes | No | 5 |
| Yousuf et al. 2021 | Yes | Yes | Yes | Yes | No | No | Yes | No | 5 |

This table is made using the Evidence Project risk of bias tool from this link: <https://systematicreviewsjournal.biomedcentral.com/articles/10.1186/s13643-018-0925-0>
