# Supplementary material for: Interventions for combating COVID-19 misinformation: A systematic realist review
Source: PLoS One. 2025 Apr 24;20(4):e0321818. doi: 10.1371/journal.pone.0321818 (PMC12021165; doi:10.1371/journal.pone.0321818)
Supplement: Attachment — Submitted filename: Realist Review Rebuttal 141024.docx [file pone.0321818.s005.docx]

University of Sussex

Department of Primary Care and Public Health

Emily Chenette

Editor-in-Chief

PLOS ONE

14 October 2024

Dear Editorial Staff,

This letter is intended to rebut the comments made on my original manuscript entitled ‘Effectiveness of digital health interventions against COVID-19 misinformation: a systematic realist review of intervention trials’ to accompany my updated manuscript in the submission process.

In the case of my original submission, the comments rightfully noted that my literature review was originally completed on 22 June 2023 and therefore represented an unacceptably outdated overview of the contemporary literature. These comments were gratefully accepted. The contemporary publication process is often extraordinarily lengthy, and alongside the rounds of feedback and editing that accompanied preparing this manuscript for submission, too much time passed without updating my manuscript.

I am happy to report that an additional, new search (taking place on 09 October 2024) has now been completed, with accompanying deselection, coding, thematic analysis, and incorporation into the original manuscript. This new search brought in 9 new papers, which brings my paper now fully up-to-date. I am additionally happy to report that these new papers, while providing significant new insights and datapoints into my analysis, also served to reaffirm and validate much of the analysis present in the original manuscript.

My systematic realist review now stands up-to-date, with substantially more and newer papers bolstering the analysis and breadth of investigation, and with underlying themes and takeaways affirmed and validated by the new literature.

I want to express my gratitude for your criticism in this matter. I believe my study is significantly improved by the inclusion of a new updating search and the eligible papers therefrom.

My paper is now the most comprehensive, validated, and up-to-date it has ever been. I hope it may meet your expectations and desires for publication in its new state. If not, I look forward to additionally insightful criticism with which I can continue to strengthen my manuscript as I seek publication.

Sincerely,

Robert Dickinson, PhD Candidate

Department of Primary Care and Public Health

University of Sussex
