# Supplementary material for: Interventions for combating COVID-19 misinformation: A systematic realist review
Source: PLoS One. 2025 Apr 24;20(4):e0321818. doi: 10.1371/journal.pone.0321818 (PMC12021165; doi:10.1371/journal.pone.0321818)
Supplement: Attachment — Submitted filename: Realist review response to reviewers PLOS ONE.docx [file pone.0321818.s006.docx]

**Response to Reviewers: Interventions for Combating COVID-19 Misinformation: A Systematic Realist Review**

Dear Reviewers,

We sincerely appreciate your time and detailed feedback on our manuscript. We have carefully addressed all concerns raised and made substantial revisions to enhance the clarity, rigor, and methodological transparency of our review. Below, we outline our responses to each point and the corresponding changes made to the manuscript.

***please note: line numbers given here refer to the manuscript with tracked changes, and will not correspond to the clean copy***

**Journal Requirements**

**1. Style Requirements**

*Comment:* “Please ensure that your manuscript meets PLOS ONE's style requirements, including those for file naming. The PLOS ONE style templates can be found at https://journals.plos.org/plosone/s/file?id=wjVg/PLOSOne_formatting_sample_main_body.pdf and <https://journals.plos.org/plosone/s/file?id=ba62/PLOSOne_formatting_sample_title_authors_affiliations.pdf>”

*Response:* We have ensured that the manuscript meets PLOS ONE's style requirements, including those for file naming. Thank you for the helpful links!

**2. Table of all studies**

*Comment:* “As required by our policy on Data Availability, please ensure your manuscript or supplementary information includes the following:

A numbered table of all studies identified in the literature search, including those that were excluded from the analyses.  For every excluded study, the table should list the reason(s) for exclusion.  If any of the included studies are unpublished, include a link (URL) to the primary source or detailed information about how the content can be accessed.”

*Response:* A numbered table of all studies identified in the literature search has been constructed. Please note: duplicated studies are shown only once in the table for clarity purposes. Reasons for exclusion are given. All included studies were published.

**3. Data extraction table**

*Comment:* “As required by our policy on Data Availability, please ensure your manuscript or supplementary information includes the following:

A table of all data extracted from the primary research sources for the systematic review and/or meta-analysis. The table must include the following information for each study: Name of data extractors and date of data extraction; Confirmation that the study was eligible to be included in the review. All data extracted from each study for the reported systematic review and/or meta-analysis that would be needed to replicate your analyses.”

*Response:* We acknowledge the importance of including a table with extracted data from the primary research sources. However, due to the nature of this review, where all text from each included study was thoroughly iterated through and qualitatively coded, compiling a full table of all extracted data would be overly extensive and not representative of the iterative process used. Instead, we refer reviewers to the Methods section, where the data extraction and coding process is described in detail. This ensures clarity and transparency while maintaining the practicality of the submission. In this instance, a full data extraction table would include all prose from all included studies.

**4. Quality assessment table**

*Comment:* “As required by our policy on Data Availability, please ensure your manuscript or supplementary information includes the following:

If applicable for your analysis, a table showing the completed risk of bias and quality/certainty assessments for each study or outcome. Please ensure this is provided for each domain or parameter assessed. For example, if you used the Cochrane risk-of-bias tool for randomized trials, provide answers to each of the signalling questions for each study. If you used GRADE to assess certainty of evidence, provide judgements about each of the quality of evidence factor. This should be provided for each outcome.”

*Response:* Table showing completed risk of bias assessments for each study has been shifted from Appendices to a supplementary information file per PLOS ONE guidelines.

**5. Missing data**

*Comment:* “An explanation of how missing data were handled.”

*Response:* Although this review does not claim perfect capture of all data relevant to this area, within the confines of the study as laid out in the methodology no missing data was encountered.

**Reviewer 1**

**1. Citations**
*Comment:* “Regarding the theoretical concerns, the introduction has many affirmations without proper citations. I won’t name each of them but I think it is remarkable that the first page includes just one citation. This of course has ramifications, and the idea that Precovid interventions against misinformation were based only in fact-checking and nudges show to some extent unfamiliarity with a literature that is broad in topics like vaccine hesitancy and HIV misinformation.”

*Response:* We have added ~20 citations to support key claims not just in the introduction but throughout the paper. Furthermore, we have toned down the referenced statement on precovid interventions and added in additional prose expanding upon the pre-covid misinformation intervention literature, beginning line 67 now reading: “The pre-COVID misinformation intervention landscape appears to focus on fact-checking [10–14]. This is not to suggest that this literature exclusively focused on fact-checking, as a rich literary body nonetheless existed examining with depth and alacrity understandings of the effectiveness and appeal of misinformation based in cognitive ability, emotional appeal, partisanship, sensationalism, and fear-mongering[15–17].”

**2. Theoretical Framework**

*Comment:* “a realist review should clarify the underlying mechanisms that explain how an intervention produces its effects and in which contexts these effects take place. My impression is that although the paper explains the different kinds of interventions evaluated, it do not provide a theoretical framework to understand the different mechanisms and contexts that produce different outcomes. The lack of a robust theoretical framework hinders the interpretation of findings.”

*Response:* We agree with this insightful suggestion, and therefore have added a theoretical framework as a new section to the manuscript. This section beginning line 115 reads as follows:

“Theoretical Framework

This study takes the theoretical framing of a realist review, focusing on uncovering the mechanisms that explain how interventions produce effects in specific contexts, working to ensure that findings can be generalized or applied to similar circumstances. These mechanisms function as the underlying processes that drive change in an intervention. They could be cognitive, as is the case for accuracy nudge interventions, where a brief reminder provides a cognitive ‘nudge’ to temporarily boost cognition when clicking on a link or news article. They could be social, as is the case for community engagement interventions, where extended and deep interaction with a community works to identify and slowly change misinformed group narratives. They could also be behavioural, as is the case for game interventions, where the act of playing a game itself is the intervention, and the interaction between player and game functions to educate and inoculate against misinformation. Table 1a in the results section provides a description of the mechanism at work within each included study for this review.

The other key piece of a realist review puzzle, contexts, are the environmental, cultural, economic, or other situational factors that influence how, why, where, and when interventions are effective. For some interventions like accuracy nudges, their use is limited to digital spaces, fitting contexts like social media platforms. For others like educational or message framing interventions, fitting contexts for use might include difficult to reach or distrustful communities. For debunking interventions, context becomes a question of time and popularity, best fitting situations where misinformation has already emerged and spread. Table 2 in the results section, alongside the wider ‘Context and Generalisability’ subsection of the results explains in greater detail the contextual nature of the interventions included in this review, as well as ranking them by generalisability.

By together clarifying the mechanisms and contexts for the interventions included, this review provides a deeper understanding of how the interventions operate and offers a theoretical contribution to realist review methods in public health and psychology. By utilising a realist review approach in the study of misinformation, where little such examination currently exists, this study contributes to the expansion of realist review approaches. Similarly, as both realist reviews and misinformation are quickly growing and relatively newly expanding areas of literature, this approach offers new insights into the study of misinformation interventions, particularly in the case of COVID-19. This review also highlights the importance of context in the countering of misinformation. By demonstrating the limited generalisability of many interventions, it contributes to the ongoing refinement of misinformation interventions to best allow bespoke policy packages of interventions to be developed best fitting the needs of the communities involved.”

**3. Polarization & Global Context**
*Comment:* “One example of this is the conclusion about addressing polarization and politics bias to understand vulnerability towards misinformation. The relationship of polarization and misinformation is now commonplace i the US literature, but it is not clear how this review raised flags about it. Moreover, it is not clear if in the literature this was a global finding or it is just applicable to the US case.”

*Response:* We found these comments helpful when considering how to justify and expand the discussion section. Firstly, we added a more explicit statement considering the wider misinformation literature compared to the narrower COVID-19 misinformation intervention literature (the focus of this study). New prose beginning line 505 reads as follows: “It is important to note that this failing is limited to the narrowly confined studies relevant to this study – the public health literature investigating COVID-19 misinformation interventions. The wider literature around misinformation has well-established links to polarisation and politics, particularly in the US.” We believe this addresses many of the concerns of the reviewer, but further agree that there is a continued question as to the global applicability of political polarization outside of the US, and therefore have amended the final direction for future research in the discussion section, beginning line 569 now reading: “4) the role of political polarisation and radicalisation in vulnerability to and the spread of misinformation, both in the United States and globally”

**4. Justification for Conclusions**
*Comment:* “Finally, the conclusions that no intervention is sufficient to counter misinformation and that games interventions are more effective should be further elaborate, to unravel which interventions are proper for which contexts and populations, as that is the ultimate goal of a realist review.”

*Response:* We have expanded our discussion to highlight why game-based interventions perform well in terms of both impact and scalability. New prose beginning line 495 reads: “Games are catching and interactive with participants, which could explain the significance found in their effect as an intervention. This interaction element could also lead to durable effects over time, although this is insufficiently studied in the current literature. Furthermore, games have the unique distinction of being fun to play, and to hold the potential to encourage public engagement with the intervention outside of experimental contexts or policies, but simple spread of an enjoyable game to play. For this same reason, games hold the potential to be a very effective subset of education interventions, where e.g. children in school could play the game as part of their curriculum attempting to build inoculation to misinformation from an early age.”

Additionally, we provide clearer guidance on which interventions may be best suited for different target populations by directing readers to the Results section devoted thereto. This prose beginning line 486 reads: “For further details on contextual fit for different interventions, please refer to the Results sub-section titled: “Context and Generalisability”.”

**5. Formatting & PRISMA Checklist**
*Comment:* “Finally, author should take into account that PlosONE have specific criteria for publication of systematic reviews (https://journals.plos.org/plosone/s/criteria-for-publication and https://journals.plos.org/plosone/s/submission-guidelines#loc-systematic-reviews-and-meta-analyses). The first problem I find with this submission is that while PRISMA flow diagram and checklist are included in the submission, the checklist items were not developed as a standalone document, but have a “location where item is reported” that do not seem to correspond with a page number of the manuscript. Also, the protocol submitted with PROSPERO should be included as supporting information, and it would be valuable to address RAMSES II standards for realist reviews. Finally, related to PlosONE criteria for publication, the manuscript does not follow the standard structure of the journal publications. For example, the methods section is presented as the last section, while in PlosONE it usually is after the introduction. This hinders comprehension, and seems like an editing error for the Study characteristic section mentions information given “before” that is actually presented at the end of the document.”
*Response:* We have restructured the manuscript to align with PLOS ONE’s formatting guidelines, including moving the Methods section earlier in the document. These adjustments were not made initially, in accordance with PLOS ONE’s guidance that formatting requirements could be waived until after the revisions stage. The PRISMA checklist has been revised for accuracy and is now provided as a standalone document with page numbers clearly indicated. As PLOS ONE requires adherence to the PRISMA standards rather than the RAMSES II standards, this study has been focused on meeting the PRISMA requirements for systematic review publication. However, we would like to note that the study follows best practices for realist reviews and has critically engaged with the RAMSES II guidelines for authors.

**Reviewer 2**

We would like to begin by thanking Reviewer 2 for their helpful comments, and particularly the comprehensive and ordered way they laid out their comments. This made for an exceptionally straightforward response and was greatly appreciated.

**1. Scope of the Review**
*Comment:* “Is the review exclusively focused on digital health interventions (i.e., internet/electronic device-based approaches)? While many of the interventions discussed in the paper include digital components, others—such as community engagement initiatives involving health promoters and outreach activities—do not appear to have a clear digital aspect. Similarly, some interventions categorized as “educational” or “message framing” may not inherently fall under the umbrella of digital health. As such, the current title of the article may inadvertently lead readers to believe that the review is limited to digital interventions. I recommend considering a title that better reflects the broader scope of the interventions analyzed.”
*Response:* We have revised the title to **"Interventions for Combating COVID-19 Misinformation: A Systematic Realist Review"** to better reflect the broader scope of interventions included.

**2. Flow Diagram Discrepancy**
*Comment:* The flow diagram indicates that 65 studies were assessed for eligibility, with 45 excluded for reasons such as lack of intervention or relevance to COVID-19. However, the original review ultimately included 26 studies, which does not align with the arithmetic presented (65 - 45 = 20). Could you clarify or revise the flow diagram to ensure consistency and transparency in the study selection process?
*Response:* We have corrected this discrepancy and ensured consistency between the text and the PRISMA diagram. While the initial diagram was meant to signify that some excluded studies were excluded for both relevance and lack of intervention, we recognise that this could be confusing for readers and so have ensured that each excluded study was excluded for one central reason. New PRISMA diagram available line 226 is as follows:

**3. Clarity and Focus on Intervention Types**

*Comment:* • Despite the review highlighting the eight types of interventions, the categorization and definitions of these types could be clearer. It is not always evident what distinguishes certain categories, and some appear to overlap conceptually. A clearer framework or typology of interventions would enhance the reader's understanding of the findings.

• The emphasis on game-type interventions is interesting, but it would be helpful to discuss why these interventions performed better in terms of impact and scalability. Are there specific features or mechanisms that make them more effective?
*Response:* We have refined the categorization of intervention types and added a summary table on line 237 to clarify distinctions between them and identify key mechanisms.

| **Intervention Type** | **Example** | **Mechanism** | **Key Findings** |
| --- | --- | --- | --- |
| Accuracy Nudges | ‘Community notes’ on Twitter | Prompts to encourage reflection on information accuracy | Mixed effectiveness, minimal impact on those heavily misinformed |
| Community Engagement | Series of workshops with community leaders | Co-created interventions tailored to community needs | Most impactful, but low scalability |
| Debunking | Fact-checking | Correcting misinformation after exposure | Moderate effectiveness, strongest among older populations |
| Prebunking | Pre-emptive messages warning about misinformation | Inoculation against misinformation before exposure | Effective, but mainly for those with healthy information habits |
| Education | A comic teaching about misinformation | Informational campaigns (videos, infographics, comics) | Highly variable impact based on preexisting knowledge levels |
| Games | Playing by taking the role of the misinformer online | Interactive misinformation training (e.g., "Bad News" game) | High impact and scalability, best for digital-literate audiences |
| Message Framing | Reshaping communication to fit different worldviews | Strategic messaging tailored to audience worldview | Effective but risk of backfire effects in certain subgroups |

**4. Quality Assessment**

*Comment:* While the review mentions quality assessment, it is not clear how the quality of the included studies influenced the synthesis of findings. The review indicates that no study was excluded based on poor quality. Were lower-quality studies given less weight, or were their limitations explicitly considered in the analysis? Clarifying this would enhance the rigor of the review.
*Response:* We appreciate the reviewer’s concern regarding the influence of study quality on the synthesis of findings. In this review, the heterogeneity of the included studies, both in terms of methodology and context, made it unfeasible to weigh studies based on their quality. Instead, the review includes all studies and addresses their limitations via quality measurement inclusion in the Study Characteristics Table as well as Appendix 1.1. This approach ensures that the findings reflect a broad spectrum of interventions, while the limitations of individual studies are acknowledged to provide context. Additionally, the division of studies by intervention type allows us to mitigate the impact of any one study's limitations by synthesizing across multiple studies within each intervention category. As such, the analysis considers all studies involved, providing a more nuanced and balanced view of the effectiveness of each intervention. Lastly, appendix 1.1 is the quality assessment table per study included. This table could be included in the main text of the manuscript if so desired. The following prose beginning line 255 has been added to the paragraph following the study characteristics table to more explicitly address these comments for readers: “Additionally, the division of studies by intervention type allows us to mitigate the impact of any one study's limitations by synthesising across multiple studies within each intervention category. Further details on quality per included study are available in Appendices Table 1.1.”

**5. Heterogeneity of Outcome Measures**
*Comment:* The review notes high heterogeneity in outcome measures and methodologies across the included studies, which complicates comparisons. While this is an important observation, the review could delve deeper into how future studies might standardize these measures or propose a framework for consistent evaluation of interventions.
*Response:* We appreciate the reviewer’s suggestion to explore how future studies might standardize outcome measures and methodologies. While this review highlights the high heterogeneity across studies, which complicates comparisons, proposing a specific framework for the consistent evaluation of interventions is beyond the scope of this review. However, we agree that standardization is an important avenue for future research and will include a brief discussion of this as a future direction in the revised manuscript. This would allow future studies to improve comparability and strengthen the evidence base for effective interventions. The new prose beginning line 570 reads: “; 5) standardisation measures or frameworks for consistent evaluation of interventions to allow for meta-analysis and quantitative comparison”

**6. Backfire Effects & Subgroup Analysis**
*Comment:* The intermittent reporting of backfire effects and subgroup-specific outcomes is a critical limitation. It would strengthen the review to provide more detail on the implications of these gaps. For instance, how might the variability in reporting backfire effects impact the development of tailored intervention packages?
*Response:* Thank you for pointing out the importance of addressing backfire effects and subgroup-specific outcomes. We agree that the intermittent reporting of these factors is a critical limitation. To address this, we recommend that the development of tailored intervention packages pay close attention to emerging research, particularly studies that delve into the role of backfire effects and subgroup-specific outcomes. In this regard, the author's upcoming study specifically focuses on identifying subgroup-specific outcomes and potential backfire effects, which will contribute to advancing this area of knowledge.

As such, we suggest that future interventions be informed by these ongoing developments in the literature, ensuring they are more robust and effective. We have added new prose in the manuscript emphasizing the need for future work in this area, beginning line 554 reading: “Given the variability in reporting backfire effects and subgroup-specific outcomes, future interventions should prioritize the development of tailored packages that account for these factors. This should be informed by emerging literature, including ongoing studies (such as the author's upcoming work), which will further investigate the role of backfire effects and subgroup-specific outcomes.”

**7. Realist Review Contribution**
*Comment:* The review mentions contributing to the expansion of realist review methodologies but does not fully explain how this contribution is achieved. Expanding on this would strengthen the review’s methodological impact.
*Response:* We now explicitly state how this review advances realist methodologies, particularly in understanding intervention-context interactions. This has been addressed in the new theoretical framework section of the manuscript as developed per this comment and comments from reviewer 1.

**8. Contextual Considerations and Policymaker Recommendations:**
*Comment:* The recommendation for policymakers to create tailored intervention packages is valuable. However, the review could benefit from more specific guidance on how to operationalize these packages. Are there concrete examples or evidence-based principles that could guide the tailoring process for different contexts and subgroups?
*Response:* While the review emphasizes the importance of tailoring intervention packages to specific contexts and subgroups, the current literature does not yet provide concrete, evidence-based principles for operationalising such packages. This gap in the literature reflects the need for further research that can develop these frameworks. The review’s recommendation serves as a call for future studies to explore the principles and practices that could guide the tailoring process, with a focus on different populations and settings. As such, a new direction for future research has been added to the manuscript, with prose beginning line 571 reading: “; and 6) policy-focused principles for the development of intervention packages to guide policymakers”

**Reviewer 3**

**1. Reader Accessibility & Definitions**
*Comment:* As a reader I would be help with a figure or “fact box” explaining all interventions available, and which are included in the analysis and together with a short example – to but everything in a context. As of now I have difficulty finding out what for example debunking really means, as some interventions are mentioned in the introduction and some are not.
*Response:* We have added an intervention-type summary table that defines each intervention type and provides brief descriptions of their mechanisms and effectiveness. This table is as follows available line 271, the same table as asked for under comment 3 of reviewer 2. Further details on intervention type and specifics of each intervention included are available in the Study Characteristics Table in the Results section.

| **Intervention Type** | **Example** | **Mechanism** | **Key Findings** |
| --- | --- | --- | --- |
| Accuracy Nudges | ‘Community notes’ on Twitter | Prompts to encourage reflection on information accuracy | Mixed effectiveness, minimal impact on those heavily misinformed |
| Community Engagement | Series of workshops with community leaders | Co-created interventions tailored to community needs | Most impactful, but low scalability |
| Debunking | Fact-checking | Correcting misinformation after exposure | Moderate effectiveness, strongest among older populations |
| Prebunking | Pre-emptive messages warning about misinformation | Inoculation against misinformation before exposure | Effective, but mainly for those with healthy information habits |
| Education | A comic teaching about misinformation | Informational campaigns (videos, infographics, comics) | Highly variable impact based on preexisting knowledge levels |
| Games | Playing by taking the role of the misinformer online | Interactive misinformation training (e.g., "Bad News" game) | High impact and scalability, best for digital-literate audiences |
| Message Framing | Reshaping communication to fit different worldviews | Strategic messaging tailored to audience worldview | Effective but risk of backfire effects in certain subgroups |

**2. Definition of "Misinformation"**
*Comment:* From what I understand, even the definition of the word misinformation is controversial, and perhaps the author’s definition should be stated in the manuscript.

*Response:* We have included a clear definition of misinformation in the introduction. The new prose beginning line 61 reads as follows: “For the purposes of this study, misinformation is broadly defined as misleading news, media content, or other information which directs viewers towards understandings of the world that do not align with a socio-politically derived consensus of legitimacy, which may be intentional or unintentional, consciously or unconsciously biased, and may or may not contain mechanisms of psychological manipulation. This definition arises out of a growing literary shift towards viewing misinformation as “that which contradicts the best expert evidence available at the time [10]”.

**3. Comparison to Other Misinformation Research**
*Comment:* I would also appreciate a discussion of how the research concerning covid-19 misinformation differ from other misinformation research.

*Response:* We appreciate the suggestion to discuss how research on COVID-19 misinformation differs from other misinformation research. However, given the scope of this review, a comprehensive discussion of these differences would be beyond its intended focus. A more detailed literature review on this topic is included in an author’s PhD thesis, which specifically explores these distinctions in depth (approximately 18,000 words). We would be happy to provide this additional context in future work or upon request, but for the current study, attempting to summarize these differences in a few hundred words would risk oversimplifying the complexities involved.

**4. Reference Placement & Completeness**
*Comment:* The references are used in an order I don’t often se, with references in the middle of a sentence and in some places an appropriate reference is missing (line 373 for example). I recommend looking over, and perhaps expanding the references.
*Response:* We have reviewed and reorganized references to ensure proper placement and completeness. A further ~20 new references have been added to address this concern. We do still, in some instances, reference in the middle of a sentence as a purposeful strategic choice to give the reader greater clarity regarding which claims are being referenced, particularly in longer sentences making multiple claims.

We appreciate the reviewers’ thoughtful and constructive feedback. We believe these revisions have significantly strengthened the manuscript, and we hope it now meets the journal's standards for publication.

Sincerely,
Robert Dickinson & Co-Authors
